# Prognostic role of CIP2A expression in serous ovarian cancer

**DOI:** 10.1038/bjc.2011.346

**Published:** 2011-09-06

**Authors:** C Böckelman, H Lassus, A Hemmes, A Leminen, J Westermarck, C Haglund, R Bützow, A Ristimäki

**Affiliations:** 1 Department of Pathology, HUSLAB and Haartman Institute, Helsinki University Central Hospital and University of Helsinki, Helsinki, Finland; 2 Genome-Scale Biology, Research Program Unit, Biomedicum Helsinki, University of Helsinki, PO Box 63 (Haartmaninkatu 8), Helsinki 00014, Finland; 3 Department of Surgery, Helsinki University Central Hospital and University of Helsinki, Helsinki, Finland; 4 Department of Obstetrics and Gynaecology, Helsinki University Central Hospital and University of Helsinki, Helsinki, Finland; 5 Centre for Biotechnology, University of Turku and Åbo Akademi University, Turku, Finland; 6 Department of Pathology, University of Turku, Turku, Finland

**Keywords:** ovarian cancer, CIP2A, survival, prognosis, grade

## Abstract

**Background:**

Cancerous inhibitor of protein phosphatase 2A (CIP2A) is an oncoprotein expressed in several solid cancers. Our purpose was to study its role in serous ovarian cancer patients, and the association to clinicopathological variables and molecular markers.

**Methods:**

We collected retrospectively 562 consecutive serous ovarian cancer patients treated at the Helsinki University Central Hospital. We stained tumour tissue microarrays for CIP2A by immunohistochemistry and constructed survival curves according to the Kaplan–Meier method. Associations to clinicopathological and molecular markers were assessed by the *χ*^2^-test.

**Results:**

We found strong cytoplasmic CIP2A immunoreactivity in 212 (40.4%) specimens, weak positivity in 222 (42.4%) specimens, and negative in 90 (17.2%). Immunopositive CIP2A expression was associated with high grade (*P*<0.0001), advanced stage (*P=*0.0005), and aneuploidy (*P=*0.001, *χ*^2^-test). Cancerous inhibitor of protein phosphatase 2A overexpression was also associated with EGFR protein expression (*P*=0.006) and *EGFR* amplification (*P*=0.043). Strong cytoplasmic CIP2A immunopositivity predicted poor outcome in ovarian cancer patients (*P*<0.0001, log-rank test).

**Conclusion:**

Our results show that CIP2A associates with reduced survival and parameters associated with high grade in ovarian cancer patients, and may thus be one of the factors that identify aggressive subtype (type II) of this disease.

Ovarian cancer is the sixth most common cancer in women and the second most common gynaecological malignancy in the world. Incidence rates have increased slowly in developed countries, with an incidence rate of 9 per 100 000 (Parkin *et al*, 2005). Five-year survival is less than 50% for ovarian cancer patients, as most cases are found at an advanced stage (Jemal *et al*, 2008). The standard treatment is extensive surgery usually followed by chemotherapy.

Ovarian surface epithelium and tubal tissue have been proposed to represent the origins of ovarian cancer, of which the most frequent subtype is serous carcinoma (Dubeau, 2008). Ovarian cancer can be divided into two subgroups: type I tumours that are slowly developing low-grade serous, mucinous, endometriod, and clear-cell carcinomas, whereas type II tumours are rapidly progressing high-grade serous or undifferentiated carcinomas (Levanon *et al*, 2008). Precursor lesions of the type II ovarian cancers are poorly understood and prognosis is poor. This type of ovarian tumours also more commonly harbour mutations in the *p53* gene and show a high proliferation index (Shih and Kurman, 2004; Landen *et al*, 2008).

Cancerous inhibitor of protein phosphatase 2A (CIP2A) is a human oncoprotein overexpressed in head and neck squamous cell carcinoma and in colon cancer (Junttila and Westermarck, 2007; Junttila *et al*, 2007; Mumby, 2007). CIP2A interacts with PP2A and prevents PP2A-mediated dephosphorylation of the oncogene c-Myc (Junttila *et al*, 2007). CIP2A is a marker of reduced overall survival in certain subgroups of gastric cancer (Khanna *et al*, 2009), and its expression associates with high grade and lymph node metastasis in breast cancer (Come *et al*, 2009). Its role in ovarian carcinogenesis is, however, unknown. To address the role of CIP2A in ovarian cancer, we investigated the association of CIP2A protein expression to clinicopathological variables and molecular markers in serous ovarian cancer.

## Patients and methods

### Patients

We collected 562 consecutive patients treated by gynaecological oncologists for serous ovarian carcinoma at the Department of Obstetrics and Gynaecology at the Helsinki University Central Hospital in 1964–2000 (median 1994). The study was approved by the National Supervisory Authority of Welfare and Health. Originally, a gynaecological pathologist examined all specimens, and in addition, another gynaecological pathologist (RB) reviewed them. Staging of the tumours was carried out according to FIGO classification, and grading according to a three-tiered grading system (Silverberg, 2000). Survival data were obtained from patient records and the Population Registry.

Tumour specimens for this study were obtained from primary surgery, and patients received no neoadjuvant treatment. Radical surgery was adopted at the end of 1980s. In 451 of 562 patients (80%), total abdominal hysterectomy and bilateral salpingo-oophorectomy were performed along with surgical removal of tumour masses, together with pelvic and/or para-aortic lymphadenectomy in 175 of these. In all, 54 (10%) patients underwent uni- or bilateral salpingo-oophorectomy, and in 57 (10%) only biopsies were obtained. Before 1990, all patients received chemotherapy according to current praxis. After 1990, all patients except those with stage 1a and b and grade 1 and 2 disease received chemotherapy (Young *et al*, 1990). Platinum-based chemotherapy served as part of first-line treatment in 404 (72%) cases together with taxanes in 194 (35%).

Response to therapy was evaluated after the initial six cycles of chemotherapy. For those who had no chemotherapy, the evaluation was performed 5–6 months post-surgery. In total, 178 (32%) patients underwent a second-look operation for evaluation of response to treatment (Miller *et al*, 1981), whereas the rest was based on gynaecological examinations, pelvic ultrasonography, CA-125 measurements, and radiological findings.

Ovarian carcinoma-specific overall survival was calculated from the date of diagnosis to death from ovarian carcinoma. Ovarian carcinoma progression-free survival was calculated for patients who were disease-free after primary treatment (surgery and first-line chemotherapy, if given) from the date of diagnosis to relapse of disease. Median age at diagnosis was 60 years (range 18–92) and median follow-up of patients alive at study end was 8.8 years (range 0.1–41.3). Five-year overall survival rate for the whole cohort was 41.2% (95% confidence interval (CI) 37.1–45.7%).

### Preparation of tumour tissue microarrays

Four representative 0.8 mm cores of tumour areas were obtained for each patient using a tissue microarray instrument (Beecher Instruments, Silver Spring, MD, USA), as described (Kononen *et al*, 1998; Kallioniemi *et al*, 2001; Torhorst *et al*, 2001).

### Immunohistochemistry

For the detailed immunohistochemistry protocol, see Khanna *et al* (2009). A rabbit polyclonal CIP2A antibody at a dilution of 1 : 10 000 for 1 h at room temperature served as the primary antibody (Soo Hoo *et al*, 2002). For validation, we stained a subset (*n*=95) with an alternative anti-CIP2A antibody (rabbit polyclonal NB100-74663, 1 : 500; Novus Biologicals, Littleton, CO, USA) according to the protocol described. Immunohistochemical analysis for p53 (monoclonal DO-7 antibody, 1 : 100; Dako, Glostrup, Denmark; Lassus *et al*, 2003), Ki-67 (polyclonal A0047 antibody, 1 : 150; Dako; Lassus *et al*, 2004), EGFR (mouse monoclonal NCL-EGFR, 1 : 150; Novocastra Laboratories, Newcastle, UK; Lassus *et al*, 2006), as well as flow cytometry (Jahkola *et al*, 1998; Lassus *et al*, 2006) have been described previously.

### Scoring of immunoreactivity

Tumour specimens were scored from tissue microarrays independently by CB and AH, blinded to clinical status and outcome data. All specimens were scored and analysed separately for cytoplasmic and nuclear CIP2A immunoreactivity. Cytoplasmic CIP2A immunopositivity was scored 0–3 according to the intensity of cancer cell immunoreactivity, and the highest intensity of the four cores was regarded to represent the final score. Completely negative immunoreactivity was scored as 0 (*n*=90) and diffuse weak cytoplasmic positivity was 1 (*n*=222). Moderately positive or focally strongly positive intensity was scored as 2 (*n*=167) and homogeneously strong intensity was 3 (*n*=45). Nuclear immunoreactivity was scored as negative (score 0) when <10% of the nuclei stained positive and as positive (score 1) when ⩾10% of the nuclei were positive. Specimens with discordant scores underwent re-evaluation with a multiheaded microscope, and the consensus score served for further analysis. In all, 524 (93%) specimens were scored successfully for CIP2A. In the final analysis, cytoplasmic immunoreactivity was analysed as negative (score 0), weakly positive (score 1), and strongly positive (scores 2 and 3), whereas nuclear immunoreactivity was analysed as negative (score 0) *vs* positive (score 1).

### Cell culture

CaOV3, OVCAR-3 (both from American Type Culture Collection, Manassas, VA, USA), and OV-4 (kindly provided by Dr Timothy J Eberlein, Harvard Medical School, Boston, MA, USA) ovarian adenocarcinoma cell lines were cultured in RPMI-1640 supplemented with 10% foetal calf serum, 2 mM L-glutamine, and antibiotics (Bio Whittaker Europe, Verviers, Belgium), and maintained at 37 °C at 5% CO_2_ in air.

### Protein extraction and immunoblot analysis

Total proteins were extracted in hot Laemmli sample buffer, whereas cytoplasmic and nuclear fractions were prepared with NE-PER nuclear and cytoplasmic extraction kit (Pierce Biotechnology Inc., Rockford, IL, USA). For western blot analysis, 30 *μ*g protein extracts were separated by 12% SDS–PAGE and transferred to nitrocellulose membranes. Membranes were blocked with 5% non-fat milk in TBS–0.1% NP40, and then incubated with the rabbit polyclonal anti-CIP2A (1 : 5000, room temperature, 1 h; Soo Hoo *et al*, 2002) or goat polyclonal anti-*β*-actin (1 : 1000, room temperature, 1 h; Santa Cruz Biotechnology, Santa Cruz, CA, USA) antibodies. Subsequently, membranes were incubated with horseradish peroxidase conjugated to goat anti-rabbit (1 : 500; Pierce Biotechnology Inc.) or donkey anti-goat (1 : 2000; Santa Cruz) for 1 h at room temperature. The proteins were visualised with SuperSignal West Femto Maximum Sensitivity Substrate (Pierce) or Proteome Grasp ECL Kit (Pierce).

### Statistical analysis

The association between CIP2A immunopositivity and clinicopathological variables was assessed by the *χ*^2^-test. The correlation between the two different CIP2A antibodies was assessed by the Spearman correlation test. Survival curves were constructed according to the Kaplan–Meier method and were compared with the log-rank test (StatView version StatView for Mac, version 5.0.1; SAS Institute Inc., Cary, NC, USA and SPSS version 17.0 for Mac; SPSS Inc., Chicago, IL, USA). For multivariate survival analysis, the Cox proportional hazard model had the following categorical covariates entered in a backward stepwise manner: FIGO stage (I, II, III, and IV), grade (1, 2, and 3), age at diagnosis (<60 and ⩾60 years), residual tumour size (⩽1 and >1 cm) and cytoplasmic CIP2A expression.

## Results

### Immunohistochemistry

**Figure 1:**
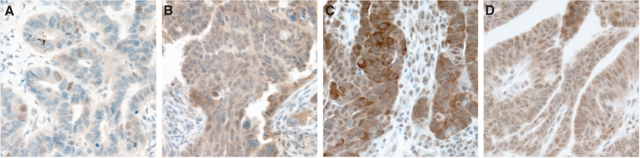
Cytoplasmic CIP2A expression in ovarian cancer specimens was scored as (**A**) negative, (**B**) weakly positive, and (**C**) strongly positive. Nuclear CIP2A expression was scored as negative (**A**) or positive (**D**). Original magnification was × 200.

We evaluated CIP2A expression separately for cytoplasmic and nuclear immunoreactivity in 562 serous ovarian cancer specimens, of which 524 (93%) were scored successfully. We found strong cytoplasmic immunopositivity for CIP2A in 212 (40.4%), weak positivity in 222 (42.4%), and negative immunoreactivity in 90 (17.2%) specimens. The cytoplasm of stromal cells remained generally negative. Nuclear CIP2A immunoreactivity was positive in 307 (58.6%) and negative in 217 (41.4%) cases (Figure 1). For validation of the CIP2A antibody, we studied cytoplasmic immunoreactivity with an alternative antibody (NB100-74663; Novus Biologicals), and found a positive correlation between cytoplasmic CIP2A immunopositivity recognised by these two antibodies (*r*_S_=0.362, *n*=95, *P*<0.0001, Spearman's correlation test).

### Association to clinicopathological variables and biomarkers

The associations between clinicopathological variables and CIP2A cytoplasmic and nuclear immunoreactivity are shown in Table 1. Patients with advanced stage (*P*=0.0005), high grade (*P*<0.0001), and ascites (*P*=0.004, *χ*^2^-test) presented more frequently with cytoplasmic CIP2A positivity (scores 1–3). CIP2A nuclear positivity was more frequent in young (*P=*0.015), low-stage patients (*P=*0.023), in those with low grade (*P*<0.0001), and in patients free from ascites (*P=*0.049).

We noted aberrant p53 immunoreactivity (*P*<0.0001), high proliferation index (Ki-67, *P*<0.0001), and aneuploidy (*P=*0.0007) to associate with cytoplasmic CIP2A expression (Table 2). CIP2A associated also with EGFR protein overexpression (*P=*0.006) and *EGFR* gene amplification (*P=*0.043).

### Survival analyses

**Figure 2:**
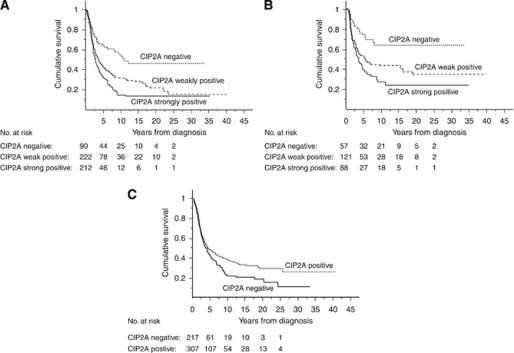
CIP2A expression and survival in ovarian cancer patients. (**A**) Overall ovarian cancer-specific survival according to the Kaplan–Meier method (*P*=0.0001, log-rank test) and (**B**) progression-free survival (*P*=0.007, log-rank test) in relation to cytoplasmic CIP2A expression in serous ovarian cancer patients. (**C**) Overall ovarian cancer-specific survival for nuclear CIP2A expression (*P*=0.013, log-rank test).

Strong cytoplasmic CIP2A positivity indicated a reduced ovarian cancer-specific 5-year survival of 31.6% (95% CI 24.7–38.4), compared to patients with weak CIP2A positivity with a 5-year survival of 42.4% (95% CI 35.6–49.2), and to those who were negative for cytoplasmic CIP2A 5-year survival of 63.0% (95% CI 52.7–73.3; *P*=0.0001, log-rank test; Figure 2A). Results were similar for progression-free survival, with 5-year survivals of 39.8% (95% CI 28.8–50.7) for CIP2A strongly positive patients, 52.1% (95% CI 42.9–61.3) for patients with weak CIP2A positivity, and 73.9% (95% CI 62.0–85.8) for CIP2A negative (*P=*0.0007, log-rank test; Figure 2B). CIP2A nuclear-negative patients had a 5-year ovarian cancer-specific survival of 37.2% (95% CI 30.3–44.2), whereas it was 45.0% (95% CI 39.2–50.8) for those who showed nuclear positivity (*P=*0.013, log-rank test; Figure 2C).

**Figure 3:**
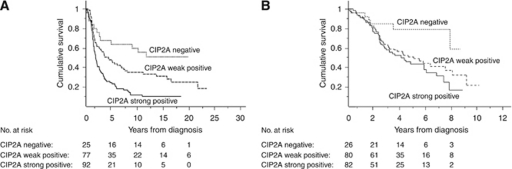
Stratified survival analyses according to adjuvant treatment. (**A**) Overall ovarian cancer-specific survival for patients who received platinum-based chemotherapy in combination with other chemotherapeutics than taxanes (*P*<0.0001, log-rank test) and (**B**) for patients who where treated with the currently used platinum-based chemotherapy combined with taxanes (*P*=0.0241, log-rank test).

Next, we stratified the survival analysis according to different adjuvant protocols. The 5-year survival for patients who received platinum-based chemotherapy combined with chemotherapeutics other than taxanes (*n*=194) was 25.2% (95% CI 16.2–34.2) for CIP2A strongly positive patients, 48.3% (95% CI 36.9–59.7) for weakly positive and 64.0% (95% CI 45.2–82.8) for CIP2A negative (*P*<0.0001, log-rank test; Figure 3A). Among patients who received the currently used platinum-based chemotherapy combined with taxanes (*n*=188), the 5-year survival for CIP2A strongly positive patients was 43.3% (95% CI 30.6–56.0), 50.8% (95% CI 38.8–62.8) for weakly positive, and 79.0% (95% CI 62.1–95.9) for CIP2A negative (*P=*0.0241, log-rank test; Figure 3B).

### Multivariate survival analysis

We performed multivariate survival analysis for, in this material, previously independent prognostic factors (age, grade, stage, residual tumour size, and aberrant p53 immunoreactivity) (Lassus *et al*, 2003; Erkinheimo *et al*, 2004). When we included cytoplasmic CIP2A expression into Cox multivariate analysis, the hazard ratio, with CIP2A-negative patients as reference, was 1.31 (95% CI 0.89–1.95) for CIP2A weakly positive and 1.20 (95% CI 0.80–1.79) for strongly positive (*P=*0.358).

### Cytoplasmic and nuclear CIP2A expression

**Figure 4:**
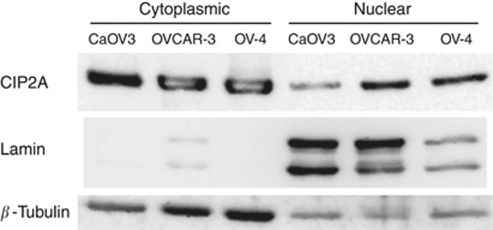
CIP2A protein expression in ovarian cancer cells. In western blot analysis, CIP2A protein is expressed both in cytoplasmic and nuclear protein fractions in the CaOV3, OVCAR-3, and OV-4 ovarian adenocarcinoma cells. Lamin was used as nuclear-positive control and *β*-tubulin as cytoplasmic-positive control.

The cellular sublocalisation of CIP2A protein was studied in the ovarian adenocarcinoma cell lines CaOV3, OVCAR-3, and OV-4. We noted that CIP2A protein is highly expressed in both the cytoplasmic and nuclear compartments (Figure 4).

## Discussion

In this study, we found that strong cytoplasmic expression of CIP2A in ovarian cancer patients is a marker of reduced overall and progression-free survival. This is in line with our previous results in gastric cancer, where we showed that CIP2A expression associates with reduced survival in the subgroups of small tumours and p53-immunopositive tumours (Khanna *et al*, 2009), and in tongue cancer, where we demonstrated that CIP2A serves as an independent marker of reduced survival (Böckelman *et al*, 2011). Dong *et al* (2010) have similarly demonstrated that CIP2A expression associates with reduced survival non-small-cell lung cancer, which was not, however, the case in another study focused on breast cancer (Come *et al*, 2009). In multivariate survival analysis in our current serous ovarian cancer material, CIP2A expression did not demonstrate independent prognostic value. All previous studies concerning CIP2A expression in tumours have focused on cytoplasmic expression. We scored cytoplasmic and nuclear expression separately and found that for nuclear CIP2A expression, the results with regard to survival were opposite compared with cytoplasmic expression, as negative nuclear expression of CIP2A indicated poor outcome. When CIP2A was first recognised as p90, Soo Hoo *et al* (2002) demonstrated its localisation to the perinuclear regions of the cytosol. Junttila *et al* (2007) noted its overexpression with predominant cytoplasmic localisation and only weak nuclear expression in head and neck squamous cell carcinoma and colon cancer. Recent studies have only addressed the cytoplasmic role of CIP2A, and the biological function of nuclear CIP2A is largely unknown. This raises an important issue, which calls for studies about the functional significance of nuclear CIP2A. In ovarian cancer cell lines, CIP2A protein was expressed to a high extent in both cytoplasmic and nuclear protein fractions. Our findings suggest that nuclear CIP2A protein may have a, to date not yet determined, functional role in ovarian carcinogenesis.

Previous studies have suggested distinct molecular pathogenesis and clinical manifestation for different histological types (Kobel *et al*, 2008), and hence, we decided to limit our study to serous histological type. Our clinical material is relatively large with a long follow-up time, unfortunately reflected by our patients being treated with heterogeneous treatment modalities. A significant proportion of the patients (*n*=188), however, received the currently used platinum-based therapy in combination with taxanes. We found that also among these patients, CIP2A was a marker of poor outcome, demonstrating that the prognostic role of CIP2A is maintained also in the patient subgroup receiving current adjuvant treatments.

Ovarian cancer has been proposed to evolve through two distinct molecular pathways: type I low-grade pathway tumours have a 5-year survival of 55% and have frequently activating mutations of *BRAF* or *KRAS*, whereas type II high-grade pathway tumours with a 5-year survival of only 30% are characterised by inactivating mutations of p53. According to this hypothesis, hallmarks of the type II pathway are high grade, high proliferation index, and p53 mutations (Shih and Kurman, 2005; Singer *et al*, 2005). In this patient material, we have previously shown that aberrant p53 expression is an independent predictor of poor survival and that it is associated with clinicopathological indicators of aggressive tumour behaviour (Lassus *et al*, 2003). Interestingly, the 5-year survival for patients with cytoplasmic CIP2A overexpressed (32%) was similar to the 5-year survival of the type II ovarian tumours (30%). We found here that cytoplasmic CIP2A positivity was associated with aggressive disease characteristics, namely high grade, advanced stage, high proliferation index, aneuploidy, and aberrant p53 immunoreactivity. Similarly, CIP2A expression was associated with high proliferation index and aneuploidy in our study on gastric cancer (Khanna *et al*, 2009), and in breast cancer, it was reported to associate with proliferation index, p53 mutation, and high tumour grade (Come *et al*, 2009). Taken together, these results propose that cytoplasmic CIP2A expression is a marker of a rapidly growing and aggressive disease.

Zhao *et al* (2010) investigated the role of *cagA*-positive *Helicobacter pylori* on CIP2A expression in gastric cancer, and found that the CagA-induced upregulation of CIP2A is mediated through the MEK/ERK pathway. Khanna *et al* (2011) continued this hypothesis by showing that the MEK1/2 and EGFR inhibitors inhibit CIP2A expression, whereas activation of MEK1/2–ERK signalling pathway stimulates CIP2A expression. They established the ETS1 transcription factor as the mediator of the EGFR–MEK1/2–ERK-induced positive regulation of CIP2A. Our association of CIP2A expression with EGFR protein expression and *EGFR* gene amplification could provide one putative mechanism for the regulation of CIP2A in human ovarian cancer.

In conclusion, our results demonstrate that overexpression of cytoplasmic CIP2A in serous ovarian cancer serves as an indicator of poor overall and progression-free survival. Associating with markers of aggressive disease, it may play a role in the type II serous ovarian cancer pathway.

## Figures and Tables

**Table 1:** Association of CIP2A with clinicopathological variables in 524 serous ovarian carcinoma patients

**Clinicopathological variable**		**Negative cytoplasmic CIP2A**		**Positive cytoplasmic CIP2A** a			**Negative nuclear CIP2A**		**Positive nuclear CIP2A**		
	***n***	***n***	**%**	***n***	**%**	***P*-value** b	***n***	**%**	***n***	**%**	***P*-value** b
*Age (years)*											
<60	260	49	19	211	81	0.314	94	36	166	64	0.015
⩾60	264	41	16	223	84		123	47	141	53	
											
*Stage*											
I	81	26	32	55	68	0.0005	25	31	56	69	0.023
II	62	5	8	57	92		21	34	41	66	
III	294	44	15	250	85		139	47	155	53	
IV	83	13	16	70	84		32	39	51	61	
											
*Grade*											
1	125	51	41	74	59	<0.0001	29	23	96	77	<0.0001
2	157	21	13	136	87		65	41	92	59	
3	242	18	7	224	93		123	51	119	49	
											
*Size (cm)*											
<10	185	25	14	160	86	0.108	77	42	108	58	0.955
⩾10	336	64	19	272	81		139	41	197	59	
											
*Residual tumour (cm)*											
<1 (optimal debulking)	196	38	19	158	81	0.096	78	40	118	60	0.224
⩾1 (suboptimal debulking)	291	40	14	251	86		132	45	159	55	
											
*Ascites*											
No	152	37	24	115	76	0.004	53	35	99	65	0.049
Yes	371	52	14	319	86		164	44	207	56	

Abbreviation: CIP2A=cancerous inhibitor of protein phosphatase 2A.

a Scores 1–3.

b *χ*^2^-test.

**Table 2:** Association of CIP2A with molecular biomarkers in 524 serous ovarian carcinoma patients

		**Negative cytoplasmic CIP2A**		**Positive cytoplasmic CIP2A** a		
**Biomarker**	***n***	***n***	**%**	***n***	**%**	***P*-value** b
*p53*						
Normal	168	55	33	113	67	<0.0001
Aberrant	341	32	19	309	91	
						
*Ki-67 (%)*						
0–10	173	52	30	121	70	<0.0001
10–25	120	10	8	110	92	
>25	91	5	5	86	95	
						
*DNA index*						
Diploid	179	43	24	136	76	0.0007
Aneuploid	173	18	10	155	90	
						
*EGFR IHC*						0.006
Normal expression	346	69	20	277	80	
Increased expression	83	6	7	77	93	
						
*EGFR* CISH						0.043
Normal (2 copies)	99	18	18	81	82	
Increased (3–5 copies)	128	14	11	114	89	
Amplified (>5 copies)	36	1	3	35	97	

Abbreviations: CIP2A=cancerous inhibitor of protein phosphatase 2A; CISH=chromogenic *in situ hybridisation*; EGFR=epidermal growth factor receptor; IHC=immunohistochemistry.

a Scores 1–3.

b *χ*^2^-test.
